# Impact of Post-Stroke Recanalization on General and Upper Limb Functioning: A Prospective, Observational Study

**DOI:** 10.3390/neurolint13010005

**Published:** 2021-02-04

**Authors:** João Paulo Branco, Filipa Rocha, João Sargento-Freitas, Gustavo C. Santo, António Freire, Jorge Laíns, João Páscoa Pinheiro

**Affiliations:** 1 Physical and Rehabilitation Medicine Department, Centro Hospitalar Universitário de Coimbra, 3030-075 Coimbra, Portugal; cr.filipa@gmail.com (F.R.); jorgelains@sapo.pt (J.L.); reabmedica@hotmail.com (J.P.P.); 2 Faculty of Medicine, University of Coimbra, 3030-075 Coimbra, Portugal; jsargentof@hotmail.com (J.S.-F.); gustavo.mtato@gmail.com (G.C.S.); afreireg@hotmail.com (A.F.); 3 Neurology Department, Centro Hospitalar Universitário de Coimbra, 3030-075 Coimbra, Portugal

**Keywords:** stroke, recanalization, rehabilitation, functioning, upper limb, functionality

## Abstract

The objective of this study is to assess the impact of recanalization (spontaneous and therapeutic) on upper limb functioning and general patient functioning after stroke. This is a prospective, observational study of patients hospitalized due to acute ischemic stroke in the territory of the middle cerebral artery (*n* = 98). Patients completed a comprehensive rehabilitation program and were followed-up for 24 weeks. The impact of recanalization on patient functioning was evaluated using the modified Rankin Scale (mRS) and Stroke Upper Limb Capacity Scale (SULCS). General and upper limb functioning improved markedly in the first three weeks after stroke. Age, gender, and National Institutes of Health Stroke Scale (NIHSS) score at admission were associated with general and upper limb functioning at 12 weeks. Successful recanalization was associated with better functioning. Among patients who underwent therapeutic recanalization, NIHSS scores ≥16.5 indicate lower general functioning at 12 weeks (sensibility = 72.4%; specificity = 78.6%) and NIHSS scores ≥13.5 indicate no hand functioning at 12 weeks (sensibility = 83.8%; specificity = 76.5%). Recanalization, either spontaneous or therapeutic, has a positive impact on patient functioning after acute ischemic stroke. Functional recovery occurs mostly within the first 12 weeks after stroke, with greater functional gains among patients with successful recanalization. Higher NIHSS scores at admission are associated with worse functional recovery.

## 1. Introduction

Stroke is a leading cause of functional disability worldwide. Most patients surviving an acute stroke present hemiparesis of brachial predominance, which constitutes a major challenge in the rehabilitation program and often leads to long-term disability [1].

Most acute strokes are of ischemic etiology [2,3] and among those, patients with the cardioembolic subtype (approximately 25%) tend to show worse clinical and functional prognosis, with higher in-hospital mortality in the acute phase and worse functioning at discharge [4,5]. Revascularization of at-risk brain tissue (ischemic penumbra area) is considered an important predictor of both clinical and functional recovery post-stroke [6]. Cell viability is maintained for a few hours after acute stroke and function can be recovered if blood flow is restored in an adequate timeframe [7,8]. The restoration of vessel patency (vascular permeability) at the site of occlusion, termed recanalization, can occur spontaneously or as a result of a therapeutic intervention. Spontaneous recanalization occurs in up to 67% of ischemic strokes, mostly within the first 48 h [9]. The factors determining spontaneous recanalization are still poorly understood, but atrial fibrillation is associated with a lower rate of spontaneous recanalization, while stage 3 hypertension is associated with higher rates of spontaneous recanalization [9].

Several therapeutic recanalization techniques—broadly classified as chemical or mechanical—have been developed to ensure reperfusion of injured brain tissue as quickly and effectively as possible [9,10]. However, different factors may limit the effectiveness of revascularization techniques. Early access to revascularization treatment is associated with better long-term functional outcomes [11], while factors such as age, severe neurological impairment, and delayed endovascular treatment are associated with worse functional prognosis even if recanalization is achieved [12].

Studies in this field have evaluated functional outcomes based on general functioning assessment tools such as the modified Rankin Scale (mRS) [11,13,14] and found recanalization to be strongly associated with improved general functional outcome [15]. These tools characterize general patient functioning but are not well-suited to assess specific domains of patients’ functioning, such as upper limb functioning. From a rehabilitation standpoint, upper limb functioning is critically important, since it will establish the ability of patients to regain autonomy. Therefore, it is important to specifically establish the impact of recanalization on upper limb functioning in addition to the global patient functioning.

This study aims to assess the impact of recanalization (spontaneous and therapeutic) on upper limb functioning and general patient functioning after ischemic stroke of the middle cerebral artery.

## 2. Materials and Methods

### 2.1. Study Design and Selection Criteria

This was a prospective, observational study of patients hospitalized due to acute ischemic stroke in a national rehabilitation center in the central region of Portugal. Patients could be included in the study if they were 18–85 years old and had acute ischemic stroke in the territory of the middle cerebral artery (confirmed through head computed tomography [CT]). Patients were excluded from participating in the study if they did not reach the emergency department (ED) within the first 3 h after symptom onset (to guarantee homogeneous reperfusion strategies according to the treatment guidelines at the time of the study), if they had signs of hemorrhagic transformation on the admission CT (graded according to the ECASS criteria [16]), if they had pre-morbid mRS >2 (as determined by interview with patients and/or direct relatives), if they showed other neurological, inflammatory, or neoplastic comorbidities, and if they did not complete head CT at admission.

After enrolment in the study, patients underwent routine clinical practice procedures for neurological and physical rehabilitation (including recanalization procedures as appropriate). Patients completed a comprehensive rehabilitation program lead by a physiatrist, based on current international guidelines for the rehabilitation of stroke patients, according to the clinical practice of the study center. The program started the second day after admission and included treatments at the nursing sector of rehabilitation, physiotherapy, and occupational therapy. Patients were followed for up to 24 weeks.

The study received favorable opinion by the Scientific Council of the Ethics Committee of the Faculty of Medicine of the University of Coimbra, Coimbra, Portugal (Ethics Committee reference identification 104-CE-2014, approved 27 October 2014). All patients or their legal representatives provided their written informed consent prior to inclusion in the study.

### 2.2. Recanalization Criteria and Procedures

On admission to the ED, patients underwent standard imaging studies including head CT and head magnetic resonance imaging (MRI) when needed. The recanalization therapies used were intravenous thrombolysis alone, thrombectomy alone or the combination of the two, following the guidelines of the European Stroke Organization [17,18,19].

In patients who underwent fibrinolysis, the evaluation of recanalization was done through transcranial doppler (TD) and recanalization was assumed for grades 4 and 5 of the thrombolysis in brain ischemia (TIBI) grading scale [20]. TD was done by the same physician for all patients.

Spontaneous recanalization was established using the same criteria among patients who did not undergo therapeutic recanalization procedures.

### 2.3. Clinical and Functional Assessment

Patients were invited to participate in the study after admission to the ED and were followed up for 24 weeks, while receiving a treatment and rehabilitation program according to routine clinical practice.

Demographic and clinical data were collected for each patient upon inclusion in the study, including age, gender, stroke subtype according to the Trial of ORG 10172 in Acute Stroke Treatment (TOAST) classification [21], severity of stroke according to National Institutes of Health Stroke Scale (NIHSS) [22], and presence of atrial fibrillation or hypertension.

Functional assessment was done using two clinically validated scales: a general functioning assessment scale, mRS, and a specific upper limb functioning assessment scale, Stroke Upper Limb Capacity Scale (SULCS) [23,24,25]. Evaluations of patient functioning (with mRS and SULCS) were completed at 4 different times: (1) 48 h, (2) 3 weeks, (3) 12 weeks, and (4) 24 weeks post-stroke. For the purposes of analysis, mRS results were dichotomized into “Low functioning” (score 3–5) and “Good functioning” (scores 0–2). SULCS scores were dichotomized into “No hand functioning” (scores 0–7) and “Good hand functioning” (scores 8–10). In this context, we opted for a dichotomization that allowed proper differentiation of distal function (i.e., if the patient had advanced hand function), since distal function is highly impactful in terms of overall functional capacity and quality of life [25].

### 2.4. Rehabilitation Interventions

During the period of the study, patients received treatment according to routine clinical practice and underwent a closely controlled rehabilitation program, following current clinical practice in the study center, which was based on current international guidelines at the time of the study [26]. During the 12 weeks, participants received inpatient care at the study center, which included 60 min of physical therapy, 30 min of occupational therapy, and 30 min of speech therapy (if aphasia was present) daily, 5 days per week. Patients who achieved maximum scores for all assessment scales and did not need further medical care, were discharged and then reevaluated at 12 weeks.

Neurological rehabilitation exercises included balance-coordination training, hand rehabilitation, stretching and relaxation exercises, walking exercises, and posture exercises. Exercises focused mainly on using the affected limb, symmetric weight bearing and transfer, mat activities, and gait training. Occupational therapy included bimanual coordination, mirror therapy, constrained induced movement therapy (if the patient fulfilled the necessary requirements) and high frequency transcranial magnetic stimulation (TMS), especially for patients with signs of depression (assessed by the physician and neuropsychologist based on the Beck depression inventory).

### 2.5. Statistical Analysis

Qualitative variables were summarized by their absolute and relative frequencies, while quantitative variables were summarized by the median, 1st and 3rd quartiles, minimum, and maximum. Mann–Whitney and Chi-square tests were used for bivariate analysis, and Monte Carlo simulations were performed where appropriate. Receiver operator characteristic (ROC) analysis was used to evaluate the discriminative power of NIHSS scores for general patient functioning and upper limb functioning. This type of analysis allows the evaluation of the relationship between a quantitative variable (NIHSS) and a categorical variable (in this case mRS and SULCS scores dichotomized as mentioned above), in order to find a cut-off point that maximizes sensibility and specificity. After recodification of the quantitative variable, estimates of specificity and sensitivity were calculated and based on these data a ROC curve was constructed. The selected cut-off point was the point corresponding to the highest Youden index (sensitivity + specificity − 1). The discriminative power of the quantitative variable (NIHSS) was, thus, quantified based on the AUC value, with higher AUC values indicating higher discriminative capacity.

All analyses were performed using IBM SPSS Statistics for Windows, Version 23.0 (IBM Corp, Armonk, NY, USA). A 5% significance level was used. Since the study was based on a convenience sample of patients admitted to the study center, no formal sample size and statistical power calculations were performed, and therefore inferential analyses should be seen as exploratory.

## 3. Results

### 3.1. Characteristics of the Study Population

During the period of the study, 115 patients were admitted with acute ischemic stroke fulfilling the selection criteria, and provided their informed consent to participate in the study. After inclusion, however, 17 patients were withdrawn from the study due to hemorrhagic transformation (*n* = 5), death (*n* = 7), and loss to follow-up (*n* = 5), thus leading to a final study population of 98 patients with 24 weeks of follow-up.

Table 1 presents the demographic and clinical characteristics of the sample. Median age was 70 years, with a slight female predominance (54.1%). Half the sample had atrial fibrillation, while hypertension was more frequent (65.3%). Most patients had stroke of cardioembolic subtype (53.1%). Most patients had occlusion in a distal middle cerebral artery segment (54.1%) and right lateralization (54.1%). The median score in NIHSS at admission was 14 (Q1: 7; Q3: 20). Overall, 69.4% of patients underwent a recanalization technique—intravenous fibrinolysis and/or endovascular thrombectomy—within a few hours after the lesion.

General functioning (measured through mRS) improved markedly in the first three weeks after stroke: from 15.3% of patients with good functioning at 48 h to 43.9% at 3 weeks. Then, general functioning plateaued at 12 weeks with 55.1% of patients showing good functioning. Upper limb functioning (measured through SULCS) showed a similar evolution over time, however the range of improvement was substantially smaller. At 48 h 17.6% of patients showed good hand functioning, improving to 38.8% at three weeks, but plateauing at approximately 44%.

Table 2 presents the level of general functioning and upper limb functioning at 12 weeks according to patients’ characteristics. Age, gender, and NIHSS at admission were significantly associated with general and upper limb functioning at 12 weeks. The segment of occlusion was also significantly associated, specifically, with upper limb functioning at 12 weeks, while for general functioning the association did not reach statistical significance. Atrial fibrillation, hypertension, stroke subtype (cardioembolic vs. all others), and importantly, the use of a recanalization technique were not significantly associated with functioning at 12 weeks.

### 3.2. Recanalization and Functioning

Recanalization was achieved in 57.1% of patients: 11.2% of patients showed spontaneous recanalization, while 45.9% of patients showed successful therapeutic recanalization (corresponding a rate of successful therapeutic recanalization of 63.4%). Table 3 explores the success of recanalization (spontaneous or therapeutic) according to the clinical characteristics of patients. The use of a recanalization technique and NIHSS score at admission were significantly associated with the success of recanalization.

Table 4 presents the evolution of patient functioning according the presence of successful recanalization and the type of recanalization (spontaneous vs. therapeutic). The proportion of patients with good general functioning was significantly higher among those with successful recanalization: 64.3% of patients with successful recanalization showed good general functioning at 24 weeks, compared to 42.9% of those without recanalization (*p* = 0.035). A similar trend was observed for upper limb functioning: 55.4% of patients with successful recanalization showed good hand functioning at 24 weeks, compared to 28.6% of those without recanalization (*p* = 0.008). Patients who achieved recanalization after a therapeutic intervention showed significantly better general functioning than those who had spontaneous recanalization at 3, 12, and 24 weeks. In terms of upper limb functioning, patients who achieved successful therapeutic recanalization also showed significant better functioning at 12 and 24 weeks, compared to those who had spontaneous recanalization.

Table 5 explores the levels of general and upper limb functioning at 12 weeks in patients with successful recanalization according to their demographic and clinical characteristics. Similarly to the overall population, among those with successful recanalization, the factors associated with good general and upper limb functioning at 12 weeks were age, gender, and NIHSS at admission. Here, however, the use of a recanalization technique was also significantly associated with better general (*p* = 0.032) and upper limb functioning (*p* = 0.041), which was not observed for the overall population.

**Figure 1:**
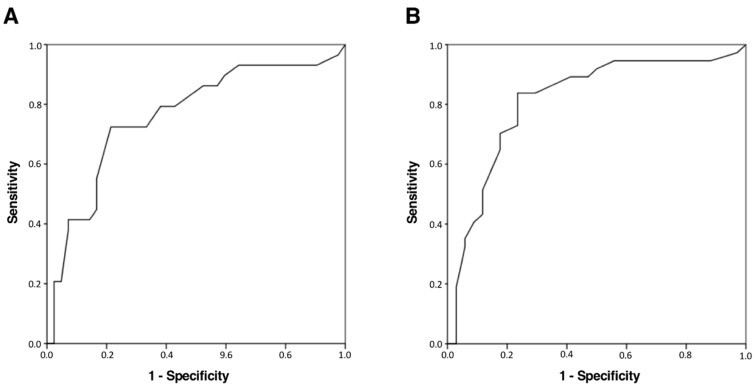
ROC curves for (**A**) low general functioning (mRS) and (**B**) no hand functioning (SULCS) at 12 weeks according to NIHSS scores at admission. mRS = modified Rankin Scale; NIHSS = National Institutes of Health Stroke Scale (NIHSS); ROC = Receiver operating characteristic; SULCS = Stroke Upper Limb Capacity Scale.

To establish the discriminative power of NIHSS scores at admission for patient functioning after stroke (12 weeks) in those patients in which a recanalization technique had been applied, ROC analysis was employed. For general functioning, NIHSS scores ≥16.5 indicate lower general functioning at 12 weeks (sensibility = 72.4%; specificity = 78.6%; AUC = 0.761 [CI 95%: 0.643–0.880], *p* < 0.001), despite the use of a therapeutic recanalization technique. For upper limb functioning, NIHSS scores ≥13.5 indicate no hand functioning at 12 weeks (sensibility = 83.8%; specificity = 76.5%; AUC = 0.812 [CI 95%: 0.706–0.918], *p* < 0.001), also despite the use of a therapeutic recanalization technique. Figure 1 presents ROC curves for both analyses.

## 4. Discussion

In this study, we assessed the impact of recanalization—both spontaneous and therapeutic—on patient functioning up to six months after acute ischemic stroke. Occurrence of successful recanalization was significantly associated with better general functioning as well as upper limb functioning from 48 h to six months after stroke. The use of therapeutic recanalization techniques was only associated with better general and upper limb functioning when recanalization was actually achieved. This is, to our knowledge, one of the few studies validating the specific impact of successful recanalization on upper limb functioning, which is of critical importance in the recovery of patient autonomy in the long-term.

The success of therapeutic recanalization has been associated with good functional prognosis in previous studies, and is one of the main predictors of good functioning at 12 weeks after stroke [11,13,14,17,23]. Intravenous thrombolysis with rt-PA achieves successful recanalization in 13% to 50% of large vessel occlusions [27], while endovascular thrombectomy achieves successful recanalization in up to 54% of cases, which is most evident in occlusions of large vessels [28,29,30]. Some studies also showed the superiority of combined treatment: mechanical thrombectomy in combination with recombinant tissue plasminogen activator (rt-PA) in large vessel occlusions [27,31]. In this study, most patients that underwent fibrinolysis and/or thrombectomy achieved successful recanalization (63.4%), a slightly higher rate than expected according to the literature. On the other hand, most patients (53.3%) that did not undergo a therapeutic recanalization technique did not show successful recanalization, as expected.

Several demographic and clinical characteristics were associated with recanalization success and patient functioning in this study. Advanced age is frequently associated with worse clinical and functional prognosis after stroke, due to frequent comorbidities and lower rehabilitation potential [32]. In this study, while increasing age was, in fact, associated with worse patient functioning, there was no discernible impact on recanalization success. A similar trend was seen for the female gender, with worse general and upper limb functioning at 12 weeks and no significant difference on recanalization success. This is consistent with previous studies [33,34]. Several factors have been suggested as potential causes of worse functioning in women, such as more serious neurological impairment presentation and lower likelihood of receiving acute stroke treatment. However, previous analysis of current evidence failed to identify the causal factors [34]. Additionally, Boheme et al. concluded that after adjustment for age, NIHSS at admission, and tPA use, the gender difference was no longer statistically significant [33]. In this study, we implemented an early and intensive rehabilitation program aimed at allowing patients to reach their full recovery potential. The use of early and high-intensity standardized rehabilitation programs has been found to significantly improve functional outcome after stroke [35]. Innovative rehabilitation interventions are currently under study—including technology-based interventions—with promising early results [36]. Such interventions could prove to be valuable tools both to address direct patient needs as well as to tackle resource constrains in healthcare systems. 

The rate of successful recanalization after administration of rt-PA is expected to be lower in patients with atrial fibrillation [32]. A recent study indicates that patients with atrial fibrillation are less likely to benefit from rt-PA, but atrial fibrillation might also lead to greater effectiveness of mechanical thrombectomy [32]. In this sample, however, we did not find atrial fibrillation to significantly impact either the rate of recanalization or the level of patient functioning. This lack of association could be because thrombus in patients with atrial fibrillation, while larger in size, tend to be easier to remove [37].

Previous studies indicate that patients with stroke of cardioembolic origin are less likely to have successful recanalization, which leads to worse overall prognosis [37,38]. In this study, however, we were not able to identify a significant impact of the cardioembolic subtype of stroke on either recanalization success or patient functioning. The lack of significant impact could conceivably be related to the dichotomization adopted (cardioembolic vs. non-cardioembolic), which does not account for variability between patients in the “non-cardioembolic” category.

Hypertension was found to influence both the occurrence of recanalization as well as the functional outcome [9,37]. In this study, however, we were not able to identify such relationships. This could be explained by the non-linear relationships identified in previous studies [9,39], which would require larger sample sizes to identify these effects. 

Occurrence of successful recanalization significantly impacted general patient functioning over the study period, which is in accordance with previous findings. Chaudhuri et al. studied functional outcome after intra-arterial fibrinolysis and found a significant improvement in general functioning at 12 weeks [14]. Bhatia et al. demonstrated that recanalization success was significantly associated to functional outcome, adding that earlier recanalization lead to significantly improved functional outcomes [11]. Gadow et al. conducted a retrospective study that included patients who underwent both recanalization techniques and demonstrated that all patients with good functioning (mRS score ≤ 2) at 12 weeks had successful recanalization [13], the criteria for recanalization were similar to those used in the present study (TICI ≥ 2b).

Successful recanalization also specifically impacted upper limb functioning as measured through SULCS. We were not able to find other studies specifically evaluating the impact of recanalization on upper limb functioning, but this is a critically important facet of patient functioning that can have great implications for patient autonomy and overall quality of life. Impaired upper limb functioning is one of the most common deficits following stroke. Upper limb functional disability affects patient independence and integration in society and, consequently, can greatly impact overall quality of life [40,41]. This study demonstrates that successful recanalization is crucial to improve upper limb functioning after stroke, which is anticipated to ultimately impact patient well-being and overall quality of life. Further studies should more specifically assess these outcomes in stroke populations, while establishing the effects of recanalization and upper limb functioning on these outcomes.

This study has several limitations. It was conducted in a highly specialized stroke unit and, therefore, this population might not be representative of other clinical care settings. While the selection criteria were considerably broad, patients with ≥85 years were excluded in an effort to avoid the bias posed by significant comorbidity and co-medication profiles, but it would be important to study this population in future studies. The sample size also introduces some limitations, especially for patients with spontaneous recanalization. The use of ultrasound to access recanalization can potentially introduce limitations in terms of accuracy and other neuroimaging modalities (such as angio-CT or angio-MRI) could provide additional data. However, these modalities would involve the need to expose the patients to more contrast agents and radiation and we wanted to avoid overexposure. The use of CT imaging is also a potential limitation to consider; this type of imaging was, nonetheless, used since it would be more representative of routine clinical practice. Collateral circulation could not be assessed with the available data, which was a limitation of the study, as it is a known modulator of outcome. Additionally, the exact topographic location of the lesion was not analyzed as not all patients had subacute MRI. However, the exact location of occlusion was assessed in all patients, ensuring adequate topographic information. There are other parameters including time metrics that could have an impact on functional outcomes given the importance of early therapeutic intervention for recanalization. These parameters were, however, not prospectively collected in this study, but should be considered in further analysis of the impact if recanalization on upper limb functioning. The potential impact of depression or cognitive impairment was also not systematically assessed in this study and could introduce bias, although patients received treatment as per routine clinical practice contributing to minimize these effects. Further studies should be designed taking into account phycological and cognitive factors, while also balancing the burden of additional assessments on patients. Finally, the instruments used to assess functionality have limitations associated with their biometric characteristics.

## 5. Conclusions

Recanalization, either spontaneous or therapeutic, has a positive impact on patient functioning after acute ischemic stroke. Functional recovery occurs mostly within the first 12 weeks after stroke, with greater functional gains among patients with successful recanalization, both in terms of general functioning and, specifically, upper limb functioning. For patients who underwent a recanalization procedure, a NIHSS score at admission ≥17 predicts low general functioning at 12 weeks (mRS 3–5) and NIHSS scores at admission ≥14 predict no hand functioning at 12 weeks (SULCS 0–7), despite the use of a recanalization technique. 

Further studies should be conducted to validate these findings in larger study populations, preferably in multicenter settings. Such studies should assess the specific impact of acute care procedures on long term general and upper limb functioning to improve treatment and rehabilitation strategies that allow stroke patients to restore their functioning to the highest degree possible.

## Figures and Tables

**Table 1:** Clinical and demographic characteristics.

Characteristics	Study Population (*n* = 98)
Age	
Median (Q1; Q3)	70.0 (62.3; 78.0)
NIHSS at admission	
Median (Q1; Q3)	14.0 (7.0; 20.0)
Gender, *n* (%)	
Male	45 (45.9)
Female	53 (54.1)
Atrial fibrillation, *n* (%)	
Yes	49 (50.0)
No	49 (50.0)
Hypertension, *n* (%)	
Yes	64 (65.3)
No	34 (34.7)
Fibrinolysis/thrombectomy, *n* (%)	
Yes	68 (69.4)
No	30 (30.6)
Stroke subtype (TOAST), *n* (%)	
Cardioembolic	52 (53.1)
Non-cardioembolic	46 (46.9)
Segment, *n* (%)	
Proximal (M1)	45 (45.9)
Distal (M2, M3)	53 (54.1)
Laterization, *n* (%)	
Right	53 (54.1)
Left	45 (45.9)
Therapeutic recanalization technique, *n* (%)	
Yes	71 (72.4)
No	27 (27.6)
Successful therapeutic recanalization, *n* (%)	
Yes	45 (63.4)
No	26 (36.6)
Type of recanalization, *n* (%)	
Spontaneous	11 (11.2)
Therapeutic	45 (45.9)
Good general functioning (mRS 0–2), *n* (%)	
48 h	15 (15.3)
3 weeks	43 (43.9)
12 weeks	54 (55.1)
24 weeks	54 (55.1)
Good hand functioning (SULCS 8–10), *n*(%)	
48 h	27 (27.6)
3 weeks	38 (38.8)
12 weeks	44 (44.9)
24 weeks	43 (43.9)

mRS = modified Rankin Scale; NIHSS = National Institutes of Health Stroke Scale (NIHSS); SULCS = Stroke Upper Limb Capacity Scale; TOAST = Trial of ORG 10172 in Acute Stroke Treatment.

**Table 2:** Patient functioning 12 weeks after stroke for the overall population, according to patients’ clinical characteristics.

	Good General Functioning (mRS 0–2)			Good Hand Functioning (SULCS 8–10)		
Characteristics	Yes (*n* = 54)	No (*n* = 44)	*p*-Value	Yes (*n* = 44)	No (*n* = 54)	*p*-Value
Age, median (Q1; Q3)	67.0 (58.5; 74.5)	74.0 (64.0; 81.0)	<0.001	67.0 (58.0; 74.0)	76.5 (66.5; 81.5)	0.003
NIHSS, median (Q1; Q3)	7.5 (6.0; 12.5)	17.0 (14.0; 23.0)	<0.001	11.0 (6.0; 16.0)	17.0 (13.0; 23.0)	<0.001
Female gender, *n* (%)	21 (38.9)	32 (72.7)	0.001	15 (34.1)	38 (70.4)	<0.001
Atrial fibrillation, *n* (%)	26 (48.1)	23 (52.3)	0.685	19 (43.2)	30 (55.6)	0.223
Hypertension, *n* (%)	33 (61.1)	31 (70.5)	0.334	27 (61.4)	37 (68.5)	0.459
Cardioembolic stroke, *n* (%)	25 (46.3)	27 (61.4)	0.137	19 (43.2)	33 (61.1)	0.077
Therapeutic recanalization technique, *n* (%)	42 (77.8)	29 (65.9)	0.191	34 (77.3)	37 (68.5)	0.335
Segment (proximal), *n* (%)	20 (37.0)	25 (56.8)	0.051	13 (29.5)	32 (59.3)	0.003

mRS = modified Rankin Scale; NIHSS = National Institutes of Health Stroke Scale (NIHSS); SULCS = Stroke Upper Limb Capacity Scale.

**Table 3:** Recanalization success, according to the patients’ clinical characteristics.

	Recanalization		
Characteristics	Yes (*n* = 56)	No (*n* = 42)	*p*-Value
Age, median (Q1; Q3)	70.0 (64.0; 78.5)	69.5 (59.0; 77.0)	0.659
NIHSS, median (Q1; Q3)	12.5 (6.50; 17.50)	17.0 (12.0; 23.0)	0.019
Female gender, *n* (%)	28 (50.0)	25 (59.5)	0.349
Atrial fibrillation, *n* (%)	27 (48.2)	22 (52.4)	0.683
Hypertension, *n* (%)	35 (62.5)	29 (69.0)	0.500
Cardioembolic stroke, *n* (%)	28 (50.0)	24 (57.1)	0.483
Therapeutic recanalization technique, *n* (%)	45 (80.4)	26 (61.9)	0.043

NIHSS = National Institutes of Health Stroke Scale (NIHSS).

**Table 4:** Patient functioning according to the occurrence of successful recanalization and the type of recanalization.

		Recanalization			Type of Recanalization		
Patient Functioning		Yes (*n* = 56)	No (*n* = 42)	*p*-Value	Spontaneous (*n*= 11)	Therapeutic (*n* = 45)	*p*-Value
Good General Functioning (mRS 0–2), *n* (%)	48 h	13 (23.2)	2 (4.8)	0.012	1 (9.1)	12 (26.7)	0.426
	3 weeks	31 (55.4)	12 (28.6)	0.008	3 (27.3)	28 (62.2)	0.048
	12 weeks	37 (66.1)	17 (40.5)	0.012	4 (36.4)	33 (73.3)	0.032
	24 weeks	36 (64.3)	18 (42.9)	0.035	4 (36.4)	32 (71.1)	0.041
Good Hand Functioning (SULCS 8–10), *n* (%)	48 h	22 (39.3)	5 (11.9)	0.003	3 (27.3)	19 (42.2)	0.498
	3 weeks	27 (48.2)	11 (26.2)	0.027	3 (27.3)	24 (53.3)	0.121
	12 weeks	32 (57.1)	12 (28.6)	0.005	3 (27.3)	29 (64.4)	0.041
	24 weeks	31 (55.4)	12 (28.6)	0.008	3 (27.3)	28 (62.2)	0.048

mRS = modified Rankin Scale; SULCS = Stroke Upper Limb Capacity Scale.

**Table 5:** Patient functioning at 12 weeks after stroke for patients with successful recanalization, according to patients’ characteristics.

	Good General Functioning (mRS 0–2)			Good Hand Functioning (SULCS 8–10)		
Characteristics	Yes (*n* = 37)	No (*n* = 19)	*p*-Value	Yes (*n* = 32)	No (*n* = 24)	*p*-Value
Age, median (Q1; Q3)	68.0 (60.0; 75.0)	79.0 (64.0; 82.0)	0.016	68.5 (61.5; 75.0)	76.5 (64.0; 82.0)	0.047
NIHSS, median (Q1; Q3)	9.0 (6.0; 14.0)	17.0 (13.0; 23.0)	0.006	7.5 (6.0; 12.0)	17.0 (13.5; 22.5)	< 0.001
Female gender, *n* (%)	13 (35.1)	15 (78.9)	0.002	9 (28.1)	19 (79.2)	< 0.001
Atrial fibrillation, *n* (%)	16 (43.2)	11 (57.9)	0.299	19 (59.4)	10 (41.7)	0.189
Hypertension, *n* (%)	22 (59.5)	13 (68.4)	0.512	20 (62.5)	15 (62.5)	1.000
Cardioembolic stroke, *n* (%)	16 (43.2)	12 (63.2)	0.158	13 (40.6)	15 (62.5)	0.105
Therapeutic recanalization technique, *n* (%)	33 (89.2)	12 (63.2)	0.032	29 (90.6)	16 (66.7)	0.041

mRS = modified Rankin Scale; NIHSS = National Institutes of Health Stroke Scale (NIHSS); SULCS = Stroke Upper Limb Capacity Scale.

## Data Availability

Datasets are available upon reasonable request from the corresponding author.

## Abbreviations

ASPECTS	Alberta Stroke Program Early CT Score
AUC	Area under the curve
CT	Computed tomography
CRP	C-reactive protein
ED	Emergency department
MRI	Magnetic resonance imaging
mRS	Modified Rankin Scale
NIHSS	National Institutes of Health Stroke Scale
S100β	S100 calcium binding protein β
rt-PA	Recombinant tissue plasminogen activator
SULCS	Stroke Upper Limb Capacity Scale
ROC	Receiver Operator Characteristic
TD	Transcranial Doppler
TIBI	Thrombolysis in Brain Ischemia
TOAST	Trial of ORG 10172 in Acute Stroke Treatment

## References

[1] Oujamaa L., Relave I., Froger J., Mottet D., Pelissier J.-Y. (2009). Rehabilitation of arm function after stroke. Literature review. Ann. Phys. Rehabil. Med..

[2] Brito R., Lins L., Almeida C., Ramos-Neto E., Araújo D., Franco C. (2014). Instrumentos de Avaliação Funcional Específicos Para o Acidente Vascular Cerebral. Rev. Neurociênc..

[3] Ward A., Payne K.A., Caro J.J., Heuschmann P.U., Kolominsky-Rabas P.L. (2005). Care needs and economic consequences after acute ischemic stroke: The Erlangen Stroke Project. Eur. J. Neurol..

[4] Arboix A., Alio J. (2012). Acute cardioembolic cerebral infarction: Answers to clinical questions. Curr. Cardiol. Rev..

[5] Murtagh B., Smalling R.W. (2006). Cardioembolic stroke. Curr. Atheroscler. Rep..

[6] Yeo L.L.L., Paliwal P., Teoh H.L., Seet R.C., Chan B.P.L., Liang S., Venketasubramanian N., Rathakrishnan R., Ahmad A., Ng K.W.P. (2013). Timing of recanalization after intravenous thrombolysis and functional outcomes after acute ischemic stroke. JAMA Neurol..

[7] Wallace A.N., Kansagra A.P., McEachern J., Moran C.J., Cross D.T., Derdeyn C.P. (2016). Evolution of endovascular stroke therapies and devices. Expert Rev. Med. Devices.

[8] Saver J.L. (2006). Time is brain—Quantified. Stroke.

[9] Yi Y., Ge Q., Li W., Wang Q.-H., Deng B., Liu C.-C., Zhang Z.-H., Zhang L.-L., Xu Z.-Q., Zhou H.-D. (2016). Predictive Factors for the Spontaneous Recanalization of Large and Middle Cerebral Arteries after Acute Occlusion. J. Stroke Cerebrovasc. Dis..

[10] Powers W.J., Derdeyn C.P., Biller J., Coffey C.S., Hoh B.L., Jauch E.C., Johnston K.C., Johnston S.C., Khalessi A.A., Kidwell C.S. (2015). 2015 American Heart Association/American Stroke Association Focused Update of the 2013 Guidelines for the Early Management of Patients With Acute Ischemic Stroke Regarding Endovascular Treatment: A Guideline for Healthcare Professionals From the American. Stroke.

[11] Bhatia R., Hill M.D., Shobha N., Menon B., Bal S., Kochar P., Watson D., Goyal M., Demchuk A.M. (2010). Low rates of acute recanalization with intravenous recombinant tissue plasminogen activator in ischemic stroke: Real-world experience and a call for action. Stroke.

[12] Shi Z.-S., Liebeskind D.S., Xiang B., Ge S.G., Feng L., Albers G.W., Budzik R., Devlin T., Gupta R., Jansen O. (2014). Predictors of functional dependence despite successful revascularization in large-vessel occlusion strokes. Stroke.

[13] von Gadow N., Nikoubashman O., Freiherr J., Block F., Reich A., Fesl G., Wiesmann M. (2017). Endovascular stroke treatment now and then-procedural and clinical effectiveness and safety of different mechanical thrombectomy techniques over time. Quant. Imaging Med. Surg..

[14] Chaudhuri J.R., Kumar R., Umamahesh M., Mridula K.R., Alladi S., Bandaru S. (2016). Outcome of acute ischemic stroke after intra-arterial thrombolysis: A study from India. Iran. J. Neurol..

[15] Rha J.-H., Saver J.L. (2007). The impact of recanalization on ischemic stroke outcome: A meta-analysis. Stroke.

[16] Fiorelli M., Bastianello S., von Kummer R., del Zoppo G.J., Larrue V., Lesaffre E., Ringleb A.P., Lorenzano S., Manelfe C., Bozzao O. (1999). Hemorrhagic Transformation Within 36 Hours of a Cerebral Infarct. Stroke.

[17] Balami J.S., Hadley G., Sutherland B.A., Karbalai H., Buchan A.M. (2013). The exact science of stroke thrombolysis and the quiet art of patient selection. Brain.

[18] DeMuro C., Clark M., Doward L., Evans E., Mordin M., Gnanasakthy A. (2013). Assessment of PRO label claims granted by the FDA as compared to the EMA (2006–2010). Value Health.

[19] Wahlgren N., Moreira T., Michel P., Steiner T., Jansen O., Cognard C., Mattle H.P., van Zwam W., Holmin S., Tatlisumak T. (2016). Mechanical thrombectomy in acute ischemic stroke: Consensus statement by ESO-Karolinska Stroke Update 2014/2015, supported by ESO, ESMINT, ESNR and EAN. Int. J. Stroke.

[20] Demchuk A.M., Burgin W.S., Christou I., Felberg R.A., Barber P.A., Hill M.D., Alexandrov A.V. (2001). Thrombolysis in brain ischemia (TIBI) transcranial Doppler flow grades predict clinical severity, early recovery, and mortality in patients treated with intravenous tissue plasminogen activator. Stroke.

[21] Adams H.P., Bendixen B.H., Kappelle L.J., Biller J., Love B.B., Gordon D.L., Marsh E.E. (1993). Classification of subtype of acute ischemic stroke. Definitions for use in a multicenter clinical trial. TOAST. Trial of Org 10172 in Acute Stroke Treatment. Stroke.

[22] Vanacker P., Lambrou D., Eskandari A., Maeder P., Meuli R., Ntaios G., Michel P. (2014). Improving prediction of recanalization in acute large-vessel occlusive stroke. J. Thromb. Haemost..

[23] Branco J.P., Oliveira S., Páscoa Pinheiro J., Ferreira P.L. (2017). Assessing upper limb function: Transcultural adaptation and validation of the Portuguese version of the Stroke Upper Limb Capacity Scale. BMC Sport Sci. Med. Rehabil..

[24] Yoon W., Kim S.K., Park M.S., Baek B.H., Lee Y.Y. (2017). Predictive Factors for Good Outcome and Mortality After Stent-Retriever Thrombectomy in Patients With Acute Anterior Circulation Stroke. J. Stroke.

[25] Houwink A., Nijland R.H., Geurts A.C., Kwakkel G. (2013). Functional recovery of the paretic upper limb after stroke: Who regains hand capacity?. Arch. Phys. Med. Rehabil..

[26] Winstein C.J., Stein J., Arena R., Bates B., Cherney L.R., Cramer S.C., Deruyter F., Bates B., Cherney L.R., Cramer S.C. (2016). Guidelines for Adult Stroke Rehabilitation and Recovery: A Guideline for Healthcare Professionals From the American Heart Association/American Stroke Association. Stroke.

[27] Khandelwal P., Yavagal D.R., Sacco R.L. (2016). Acute Ischemic Stroke Intervention. J. Am. Coll. Cardiol..

[28] Jiang F., Jiang Y., Zhi H., Dong Y., Li H., Ma S., Wang Y., Dong Q., Shen H., Wang Y. (2017). Artificial intelligence in healthcare: Past, present and future. Stroke Vasc. Neurol..

[29] Saver J.L., Goyal M., Van Der Lugt A., Menon B.K., Majoie C.B.L.M., Dippel D.W., Campbell B.C., Nogueira R.G., Demchuk A.M., Tomasello A. (2016). Time to Treatment With Endovascular Thrombectomy and Outcomes From Ischemic Stroke: A Meta-analysis. JAMA.

[30] Pierot L., Gawlitza M., Soize S. (2017). Techniques for endovascular treatment of acute ischemic stroke. Rev. Neurol. (Paris).

[31] Balami J.S., Sutherland B.A., Edmunds L.D., Grunwald I.Q., Neuhaus A.A., Hadley G., Karbalai H., Metcalf K.A., DeLuca G.C., Buchan A.M. (2015). A systematic review and meta-analysis of randomized controlled trials of endovascular thrombectomy compared with best medical treatment for acute ischemic stroke. Int. J. Stroke.

[32] Singer O.C., Haring H.-P., Trenkler J., Nolte C.H., Bohner G., Reich A., Wiesmann M., Bussmeyer M., Mpotsaris A., Neumann-Haefelin T. (2013). Age dependency of successful recanalization in anterior circulation stroke: The ENDOSTROKE study. Cerebrovasc. Dis..

[33] Boehme A.K., Siegler J.E., Mullen M.T., Albright K.C., Lyerly M.J., Monlezun D.J., Jones E.M., Tanner R., Gonzales N.R., Beasley T.M. (2014). Racial and gender differences in stroke severity, outcomes, and treatment in patients with acute ischemic stroke. J. Stroke Cerebrovasc. Dis..

[34] Coupar F., Pollock A., Rowe P., Weir C., Langhorne P. (2012). Predictors of upper limb recovery after stroke: A systematic review and meta-analysis. Clin. Rehabil..

[35] Yagi M., Yasunaga H., Matsui H., Morita K., Fushimi K., Fujimoto M., Koyama T., Fujitani J. (2017). Impact of Rehabilitation on Outcomes in Patients With Ischemic Stroke: A Nationwide Retrospective Cohort Study in Japan. Stroke.

[36] Hatem S.M., Saussez G., Della Faille M., Prist V., Zhang X., Dispa D., Bleyenheuft Y. (2016). Rehabilitation of Motor Function after Stroke: A Multiple Systematic Review Focused on Techniques to Stimulate Upper Extremity Recovery. Front. Hum. Neurosci..

[37] Giray S., Ozdemir O., Baş D.F., İnanç Y., Arlıer Z., Kocaturk O. (2017). Does stroke etiology play a role in predicting outcome of acute stroke patients who underwent endovascular treatment with stent retrievers?. J. Neurol. Sci..

[38] Nam H.S., Lee K., Kim Y.D., Choi H.-Y., Cho H.-J., Cha M.-J., Nam C.M., Heo J.H. (2011). Failure of complete recanalization is associated with poor outcome after cardioembolic stroke. Eur. J. Neurol..

[39] Martins A.I., Sargento-Freitas J., Silva F., Jesus-Ribeiro J., Correia I., Gomes J.P., Aguiar-Gonçalves M., Cardoso L., Machado C., Rodrigues B. (2016). Recanalization Modulates Association Between Blood Pressure and Functional Outcome in Acute Ischemic Stroke. Stroke.

[40] Wade D.T., Langton Hewer R., Skilbeck C.E., David R.M. (1985). Stroke: A Critical Approach to Diagnosis, Treatment and Management.

[41] Goulding R., Thompson D., Beech C. (2004). Caring for patients with hemiplegia in an arm following a stroke. Br. J. Nurs..

